# Minimal Optimized Effective Potentials for Density Functional Theory Studies on Excited-State Proton Dissociation

**DOI:** 10.3390/mi12060679

**Published:** 2021-06-10

**Authors:** Pouya Partovi-Azar, Daniel Sebastiani

**Affiliations:** Institute of Chemistry, Martin-Luther-University Halle-Wittenberg, Von-Danckelmann-Platz 4, 06120 Halle (Saale), Germany; daniel.sebastiani@chemie.uni-halle.de

**Keywords:** density functional theory, time-dependent density functional theory, optimized effective potentials, excited states, proton dissociation

## Abstract

Recently, a new method [P. Partovi-Azar and D. Sebastiani, *J. Chem. Phys.* 152, 064101 (2020)] was proposed to increase the efficiency of proton transfer energy calculations in density functional theory by using the T${}_{1}$ state with additional optimized effective potentials instead of calculations at S${}_{1}$. In this work, we focus on proton transfer from six prototypical photoacids to neighboring water molecules and show that the reference proton dissociation curves obtained at S${}_{1}$ states using time-dependent density functional theory can be reproduced with a reasonable accuracy by performing T${}_{1}$ calculations at density functional theory level with only one additional effective potential for the acidic hydrogens. We also find that the extra effective potentials for the acidic hydrogens neither change the nature of the T${}_{1}$ state nor the structural properties of solvent molecules upon transfer from the acids. The presented method is not only beneficial for theoretical studies on excited state proton transfer, but we believe that it would also be useful for studying other excited state photochemical reactions.

## 1. Introduction

Many fundamental chemical processes are triggered when the electrons in a system are excited. The interaction of a molecule with its environment at an electronic excited state can be largely different from that in the electronic ground state. Therefore, it has become possible to indirectly investigate the nature of such interactions as well as structural properties of solvents by studying the change in properties of molecular probes upon excitation [1,2,3,4,5]. Electronic excitations can also lead to inter- or intramolecular proton transfers [6,7,8,9,10,11]. In particular, photoinduced proton transfer in solutions is of fundamental interest in a large variety of chemical and biological applications such as energy storage systems and sensors [12,13,14,15,16,17,18].

From a theoretical standpoint, *ab initio* molecular dynamics simulation (AIMD) is a suitable tool for studying local structure as well as structural dynamics of solute and solvent molecules, taking both electronic structure and thermal effects into account [19]. Density functional theory (DFT) [20,21] is usually the method of choice for including electronic degrees of freedom in AIMD simulations at ground state, due to a reasonable balance between accuracy and computational efficiency. However, electronic excited states are not accessible in DFT and higher-level methods, such as time-dependent DFT (TDDFT) [22], equation of motion coupled cluster [23,24,25], etc., are needed. Nevertheless, force calculations using higher-level methods are prohibitively time consuming, making AIMD simulations based on these methods limited only to small system sizes and short simulation times.

In the past years, there have been various approaches to improve DFT description of proton transfer at excited states [26,27,28,29,30,31,32,33]. Recently, a method has been proposed where T${}_{1}$ state is used and further modified using effective potentials for acidic atoms to mimic the actual S${}_{1}$ state [34]. T${}_{1}$ state, unlike S${}_{1}$, is variationally accessible in DFT and therefore the proposed method allows for efficient calculation of proton transfer energies and barriers at excited state. The modification is done by adding effective potentials, which are represented via atom-centered Gaussian functions. It has been demonstrated that the proton transfer energy can be accurately obtained by optimizing one additional effective potential per non-hydrogen atoms in a photoacid. It has also been shown that in order to obtain the correct kinetics of the proton dissociation reaction, one would need to optimize an additional effective potential for the acidic hydrogen as well. Here, we explore the possibility of finding the whole dissociation curve by optimizing only one additional non-local effective potential, represented as an expansion in terms of Gaussian projectors and only for the acidic hydrogens. As target photoacids, we consider phenol, 2- and 4-cyanophenol, 1- and 2-naphthol, and 7-hydroxyquinoline.

We emphasize that in this work, the aim is solely to reproduce the proton dissociation curves of the above photoacids at their respective first excited state. Although our preliminary investigations indicate that correct excited state wavefunctions along the reaction path could, in principle, be obtained using additional effective potentials, this still needs to be studied in detail and goes beyond the scope of the present article.

## 2. Methodology

The reference calculations are performed at TDDFT level using the Orca code [35,36]. Application-oriented calculations are performed at the DFT level along with pseudopotential approximation using the CP2K software [37,38]. All-electron DFT and TDDFT calculations with Orca are carried out using the correlation-corrected cc-pVTZ basis set [39] together with ωB97X-D3 range-separated, hybrid exchange–correlation (XC) functional [40]. The ωB97X-D3 functional has been already demonstrated to produce excited-state properties, including proton dissociation curves, with very good agreement with higher-level quantum chemical methods [34]. Therefore, in this work the reference calculations are carried out using ωB97X-D3 functional at TDDFT level. In the TDDFT calculations, the Tamm–Dancoff approximation [41,42] is used, and in all excited state calculations the target electronic state is set to S${}_{1}$ while, altogether, 10 excited state roots are computed.

**Figure 1:**
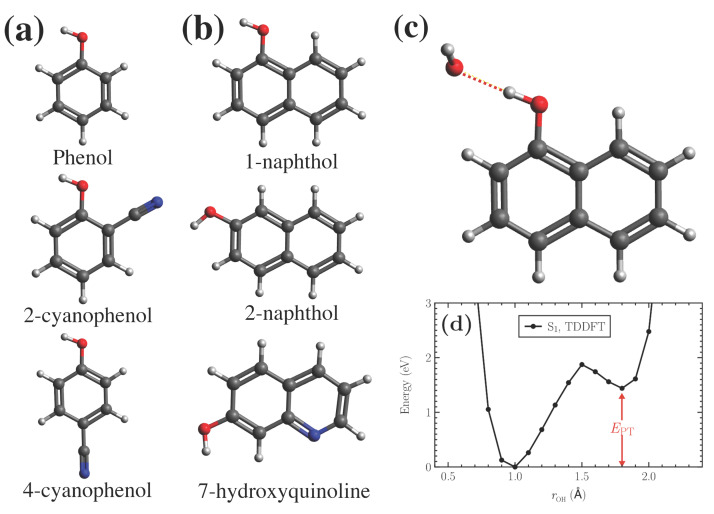
(**a**,**b**) The prototypical photoacids considered in this work. (**c**) 1-Naphthol/water complex. Similar complexes are used for each photoacid in order to calculate the respective proton dissociation curves. (**d**) Reference proton dissociation curve for the 1-naphthol/water complex in (**c**) obtained using TDDFT calculations at S${}_{1}$ state.

The proton transfer energy is defined based on dissociation of a proton from prototypical photoacids phenol, 2- and 4-cyanophenol, 1- and 2-naphthol, and 7-hydroxyquinoline (7HQ), (Figure 1a,b) to one neighboring water molecule.

The ground state structures of these complexes are first optimized in vacuum using all-electron DFT calculations with ωB97X-D3 XC functional. Afterwards, the hydrogen is gradually moved from each photoacid along the O–H⋯O line toward the water molecule with 0.1 Å strides, and the corresponding total energies are calculated (see Figure 1c,d for 1-naphthol).

As for the additional effective potentials for the acidic hydrogens, here we choose Goedecker–Teter–Hutter (GTH)-type Gaussian functions [43,44]. The analytic form of these potentials provides considerable efficiency in numerical calculations using plane-wave and mixed Gaussian/plane-wave basis sets [45,46,47]. Our additional effective potential is expressed using the non-local potential,

(1) $$\begin{array}{cc} \; & V_{\mathrm{nl}}\left(r,r^{\prime }\right)=\sum_{lm}\sum_{ij}\langle r|p_{i}^{lm}\rangle h_{ij}^{l}\langle p_{j}^{lm}|r^{\prime }\rangle ,\\ \; & \langle r|p_{i}^{lm}\rangle =N_{i}^{l}\;Y^{lm}\left(\theta ,\phi \right)\;r^{l+2i-2}\;\exp\left[-\frac{1}{2}{\left(\frac{r}{r_{l}}\right)}^{2}\right]. \end{array}$$

Here, $\langle r|p_{i}^{lm}\rangle$ are Gaussian-type projectors and $h_{ij}^{lm}$ denote the i×j symmetric coefficient matrix for the angular momentum channel *l*, while $r_{l}$ represents the corresponding effective radius of the projector. $Y^{lm}\left(\theta ,\phi \right)$ are the spherical harmonics and $N_{i}^{l}$ are normalization constants. We find that appropriate effective potentials for the acidic hydrogens have to be effective only at typical OH bond distances (covalent and H-bonded, i.e., ∼1 Å ) and longer, but not in the region of the nucleus itself. In Equation (1), we consider i=1,2 and in order to be able to impose the above condition, we set $h_{11}^{0}$ explicitly to zero and optimize three parameters, i.e., $h_{12}^{0}$ and $h_{22}^{0}$ together with $r_{0}$.

By minimizing the function

(2) $$f\left(r_{0},h^{0}\right)={\left[E_{\mathrm{ref}}^{S_{1}}-E_{\mathrm{DFT}}^{T_{1}}\left(r_{0},h^{0}\right)\right]}^{2},$$

we require that DFT values for the proton transfer energies calculated at the T${}_{1}$ state with additional effective potentials to be as close as possible to those obtained at the S${}_{1}$ state of the target complexes using TDDFT. Here, *E* denotes the proton transfer energy, $h^{0}$ is a vector with components representing projector coefficients $h_{12}^{0}$ and $h_{22}^{0}$ for the acidic hydrogen. T${}_{1}$ state is chosen as the starting electronic configuration because the previous studies have shown that it can partially capture the excited state properties of similar photoacids [10]. The minimization of the function *f* is done using gradient descent method, where the step size is updated every five steps using the Barzilai–Borwein method [48]. The starting value for the projector coefficients $h_{12}^{0}$ and $h_{22}^{0}$ is set to zero, while for $r_{0}$, we use 1.0 Å as a starting value. Additionally, we use the BLYP XC functional [49,50] in the DFT calculations along with Grimme’s dispersion correction [51] and a triple-ζ TZVP-MOLOPT basis set [38]. For dipole moment calculations at the T${}_{1}$ state with the extra optimized effective potentials, we use maximally localized Wannier functions [52].

## 3. Results and Discussion

**Figure 2:**
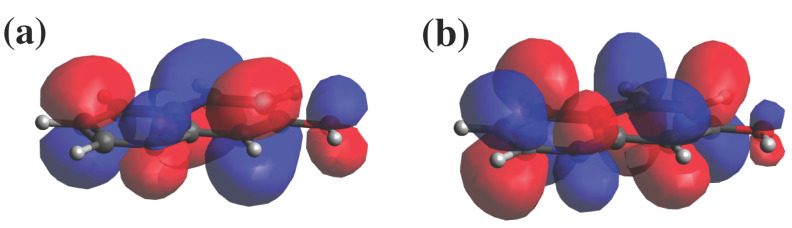
(**a**) HOMO and (**b**) LUMO orbitals of 7HQ photoacid at ground state calculated at DFT level.

Natural transition orbitals [53] show that in all the photoacids considered here, the main contribution (larger than 70% in all cases) to the S${}_{0}$→ S${}_{1}$ and S${}_{0}$→ T${}_{1}$ excitations comes from a HOMO to LUMO transition with a $\pi -\pi^{*}$ nature. As an example, the HOMO and LUMO orbitals of 7HQ at its ground state are shown in Figure 2.

We also observe that both excitations result in a depletion of charge around the acidic hydrogen.

We find that a better agreement with the reference TDDFT calculations can be reached by adapting the following optimization procedure: (i) we optimize $r_{0}$ and $h_{12}^{0}$ to obtain the correct proton transfer energies; (ii) using these optimized parameters, we optimize $h_{22}^{0}$ and again $r_{0}$ to reach the correct values for the transition energies. This procedure can be iterated until a convergence is reached. Based on our findings, typically two iterations are enough to reach the converged values.

The optimized values of $r_{0}$, $h_{12}^{0}$, and $h_{22}^{0}$ for the photoacids considered in this work are given in Table 1.

**Figure 3:**
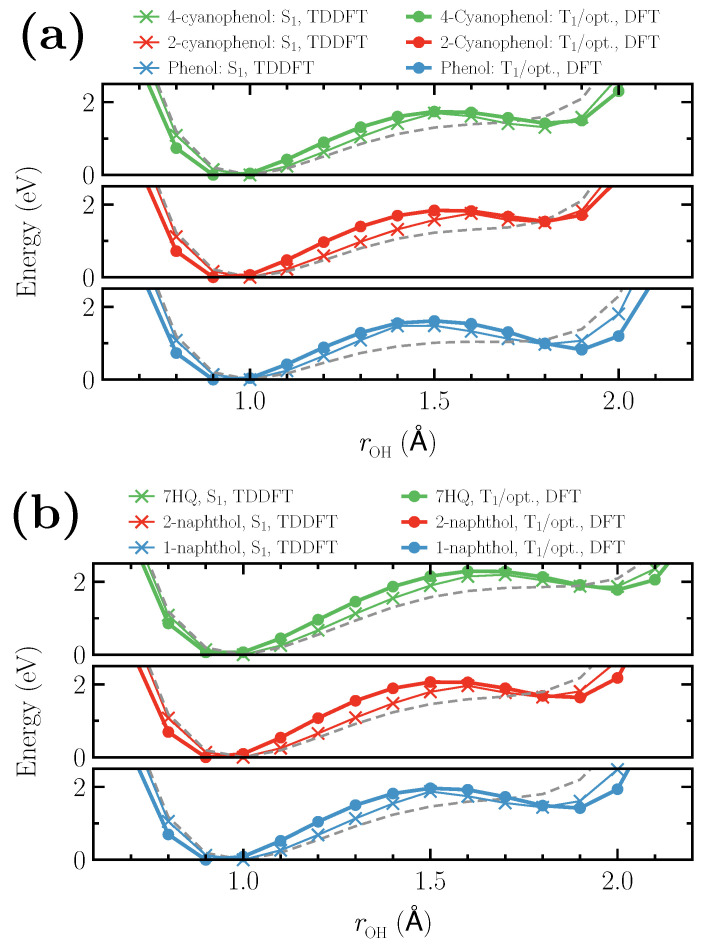
Proton dissociation curves for (**a**) phenol- and (**b**) naphthol-based photoacids. The reference data points obtained using TDDFT at S${}_{1}$ states are shown as crosses. DFT results at T${}_{1}$ state with the additional optimized effective potentials for the acidic hydrogens are shown as solid circles. Gray dashed lines show the dissociation curves obtained at T${}_{1}$ states without any additional potentials.

We also observe that the parameter values only marginally differ from the values reported in Table 1 when another XC functional is used for DFT calculations at T${}_{1}$ state. The difference is found to be less than 5% for the case of PBE functional [54]. Proton dissociation curves calculated using the parameters in Table 1 are shown in Figure 3a,b for phenol- and naphthol-based photoacids, respectively.

The reference energy calculations using TDDFT at respective S${}_{1}$ states are denoted with crosses. The dissociation curves at the T${}_{1}$ state with the optimized potentials (T${}_{1}$/opt.) are obtained after repeating the optimization steps (i) and (ii) for two iterations. Gray dashed lines show the dissociation curves obtained at respective T${}_{1}$ states without any additional potentials. The S${}_{1}$ TDDFT energies during the proton dissociation are well reproduced by DFT calculations at T${}_{1}$ state with the optimized parameters giving a mean absolute error (MAE) of ∼204 and ∼255 meV in 0.8 to 2.0 Å O⋯H distances for phenol- and naphthol-based photoacids, respectively. Restricting the error estimation only to the two minima and the transition point, MAE is calculated to be ∼55 and ∼78 meV for phenol- and naphthol-based photoacids, respectively. However, the OH covalent bond distance in the photoacids is underestimated in almost all cases (except for 7HQ) by, at most, 0.1 Å. The O⋯H distance at the transition state is correctly reproduced for 4-cyanophenol and 1-naphthol, while it is underestimated for 2-naphthol, 7HQ, and 2-cyanophenol. It is slightly overestimated for phenol. Additionally, we find that the optimized potentials do not alter the nature of the T${}_{1}$ states. In the case of 7HQ, for example, the maximum change in the atomic orbital coefficients contributing to the HOMO molecular orbital of majority spin obtained using T${}_{1}$/opt. and that computed at T${}_{1}$ state without any additional potential is found to be less than 10%. Therefore, the shape of these orbitals are almost identical in T${}_{1}$ and T${}_{1}$/opt. calculations. However, we observe a slight change in the HOMO-1 orbital in T${}_{1}$ and T${}_{1}$/opt. calculations. Our specific inspection shows that in the case of 7HQ, it is mainly caused by a ∼63% change in the *s* atomic orbital coefficient of the oxygen atom in the HOMO-1 wavefunction at T${}_{1}$/opt. calculations.

**Figure 4:**
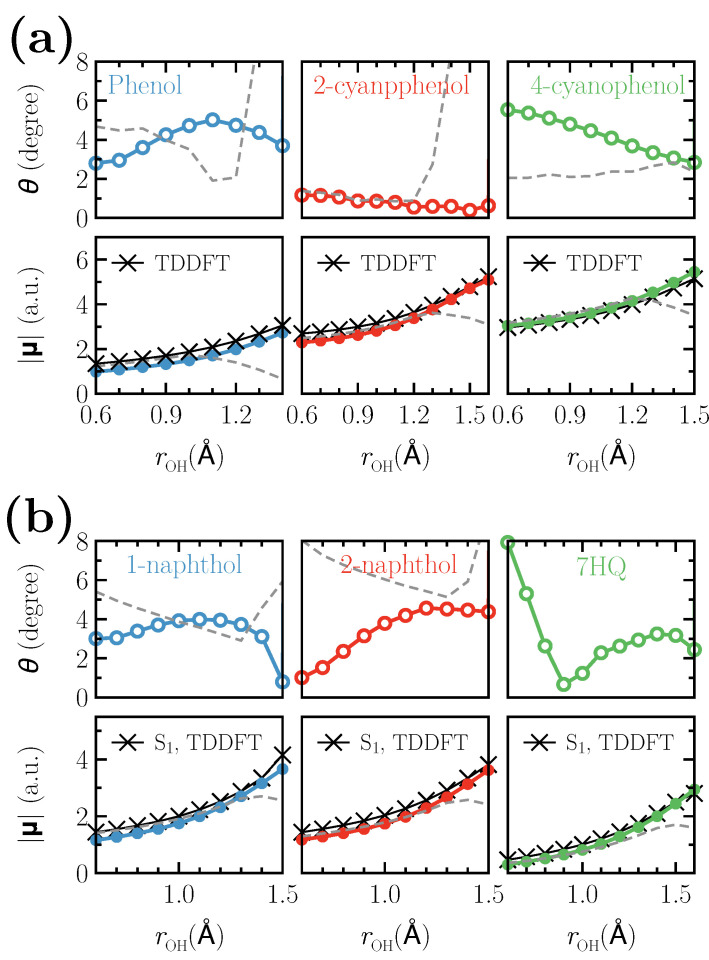
Dipole moments of (**a**) phenol- and (**b**) naphthol-based photoacids. The lower panels show the length of the dipole moment vector, while the upper panels present the angle between the dipole moment vectors calculated at T${}_{1}$ state together with the optimized effective potentials and the ones computed at S${}_{1}$ state for the same systems using TDDFT. The black crosses in the lower panels denote the reference TDDFT values. Gray dashed curves represent the values obtained at T${}_{1}$ state without any additional potential.

Additionally, to assess the performance of the optimized effective potentials for the acidic hydrogens, we calculate the molecular dipole moments as a function of $r_{OH}$ around the respective global minimum of the proton dissociation curve up to the corresponding transition distance. The calculated dipole moments are presented in Figure 4a,b. The lower panels in Figure 4 show the length of the dipole moment vectors while the upper panels present the angle between the dipole moment vectors calculated at T${}_{1}$ state together with the optimized effective potentials for the acidic hydrogens and the ones computed at S${}_{1}$ state for the same systems using TDDFT. The reference dipole moment lengths are shown in black crosses and the gray dashed lines represent the results obtained at the T${}_{1}$ state without any optimized potentials. Both the length and the direction of the dipole moments obtained in the T${}_{1}$/opt. calculations are in a very good agreement with the reference dipole moments in phenol- as well as naphthol-based photoacids in the O⋯H distances shown in Figure 4a,b. However, for longer O⋯H distances, the T${}_{1}$/opt. results become largely different from the reference data due to non-adiabatic level crossing from S${}_{1}$ to S${}_{2}$, which was found to occur in all the systems considered in this work. The eigenvalue of the S${}^{2}$ operator, i.e., 2, in both calculations remains virtually identical in the O⋯H distances shown in Figure 4.

**Figure 5:**
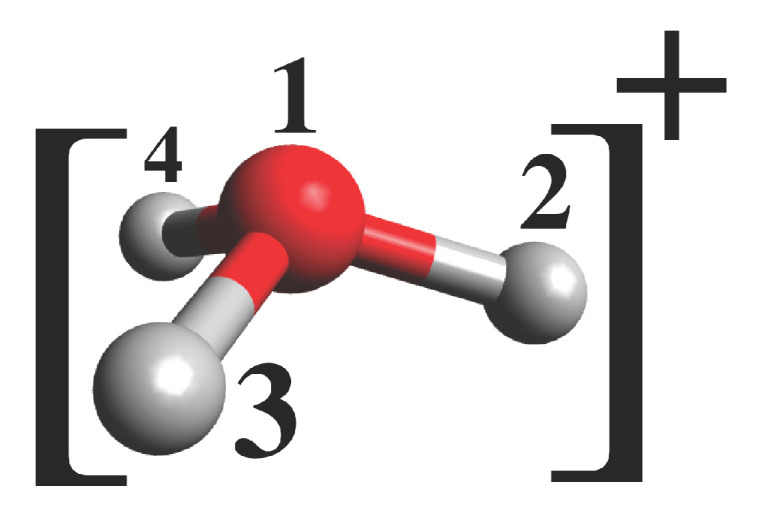
A hydronium cation which is assumed to form after proton transfer from the photoacids considered in this work. The transferred proton is denoted as 3.

Finally, we focus on a situation in which the acidic proton has transferred to the neighboring water molecule, for example, in an AIMD simulation. The aim here is to make sure that the covalent bonding to the water molecule is not changed upon transfer of the proton from the photoacids. To this end, we perform a series of geometry optimizations on a hydronium cation with one of the hydrogens carrying the extra optimized potentials (number 3 in Figure 5). The geometrical properties of such a hydronium cation are given in Table 2. The bond lengths and angles are observed to remain very close to the ones obtained for a hydronium cation without any additional potential. The biggest difference is found for phenol in terms of the angles between the bonds. This finding indicates that the effective potentials for the acidic hydrogens can not only be used to reproduce the correct energies and barriers of excited state proton transfer reactions from a photoacid to a solvent molecules but can also be utilized throughout an AIMD simulation without the need to switch it off for the solvent molecules, which usually remain at their ground electronic state.

## 4. Conclusions

In this work, we have proposed and optimized a specific form of effective potentials for acidic hydrogens to improve the efficiency of density functional theory calculations in predicting energies and barriers of proton transfer reactions at excited state. In this approach, a non-local potential of Goedecker–Teter–Hutter type, represented as an expansion in terms of Gaussian projectors, is used for acidic hydrogens in order to reconstruct the correct proton dissociation curves at S${}_{1}$ state by performing T${}_{1}$ calculations at density functional theory level. We have employed this approach to study proton dissociation from several prototypical photoacids to neighboring water molecules. We have shown that after optimizing the additional effective potentials for the acidic hydrogens, both reaction energies and barriers of the proton dissociation reactions can be reproduced with reasonable accuracy. Additionally, we have found that, in all cases considered here, the structural properties of the hydronium cation formed after the full transfer of the acidic hydrogens to the neighboring molecule is nearly identical to the one without additional potentials. This implies that the additional effective potentials can be utilized for the whole system, i.e. both photoacid and solvent molecules, without the need to switching them off after the proton transfer from the excited photoacid to the solvent. The presented method is expected to be useful for studying excited state photochemical reactions in general systems.

## Figures and Tables

**Table 1:** Parameters for the acidic hydrogens (in atomic units) optimized for excited state proton transfer calculations.

Phenol-Based Photoacids			
	$r_{0}$ (a.u.)	$h_{12}^{0}$	$h_{22}^{0}$
Phenol	2.112996	0.465280	0.460871
2-Cyanophenol	1.797464	0.094554	0.395386
4-Cyanophenol	2.002620	0.176501	0.479233
**Naphthol-Based Photoacids**			
	$r_{0}$ (a.u.)	$h_{12}^{0}$	$h_{22}^{0}$
1-Naphthol	2.019293	0.333423	0.4976893
2-Naphthol	1.979366	0.269279	0.3800635
7HQ	2.085884	0.132695	0.3663171

**Table 2:** Bond lengths and angles of a hydronium molecule with additional optimized effective potential on one proton (number 3 in Figure 5). The optimized parameters are the same as in Table 1.

From	d${}_{12}$ (Å)	d${}_{13}$ (Å)	d${}_{14}$ (Å)	*∠*213 (${}^{\circ }$)	*∠*214 (${}^{\circ }$)	*∠*314 (${}^{\circ }$)
Phenol	0.99	0.97	0.99	106	106	106
2-cyanophenol	0.99	0.97	0.99	110	109	110
4-cyanophenol	0.99	0.97	0.99	108	108	108
1-naphthol	0.99	0.97	0.99	108	107	108
2-naphthol	0.99	0.97	0.99	109	108	109
7HQ	0.99	0.97	0.99	109	109	109
No OEP	0.99	0.99	0.99	111	111	111
